# Validity and reliability of the 8-item version of the jaw functional limitation scale (JFLS-8) in the Arabic language

**DOI:** 10.22514/jofph.2026.018

**Published:** 2026-03-12

**Authors:** Ziad Azar, Christelle Haddad, Lynn Fayad, Tia Azar, Tylor Cosgrove, Mirna Fawaz, Sahar Obeid, Feten Fekih-Romdhane, Souheil Hallit

**Affiliations:** ^1^School of Medicine and Medical Sciences, Holy Spirit University of Kaslik, P.O. Box 446, Jounieh, Lebanon; ^2^School of Psychology, University of South Australia, 5000 Adelaide, SA, Australia; ^3^School of Nursing, Beirut Arab University, 1107 Beirut, Lebanon; ^4^Social and Education Sciences Department, School of Arts and Sciences, Lebanese American University, P.O. Box 36, Jbeil, Lebanon; ^5^The Tunisian Center of Early Intervention in Psychosis, Department of Psychiatry “Ibn Omrane”, Razi Hospital, 2010 Manouba, Tunisia; ^6^Faculty of Medicine of Tunis, Tunis El Manar University, 1007 Tunis, Tunisia; ^7^Applied Science Research Center, Applied Science Private University, 11931 Amman, Jordan

**Keywords:** Jaw dysfunction, Validity, Reliability, Arabic, Psychometric properties

## Abstract

**Background**: The Jaw Function Limitation Scale (JFLS) is a recently developed instrument that assesses specific jaw function limitations and objective restrictions in daily activities such as chewing, jaw mobility, and verbal communication in a valid and reliable manner. The scale has been translated and validated in several languages and contexts, but no Arabic version is currently available. To address the absence of a validated tool for assessing jaw dysfunction in Arabic-speaking populations, this study aimed to evaluate the validity, reliability, and factor structure of the 8-item version of the JFLS. **Methods**: This cross-sectional study was conducted between August and October 2025 and included 427 Arabic-speaking adults from Lebanon. Confirmatory factor analysis (CFA) was used to validate the scale. **Results**: The analysis supported a three-factor structure. Internal reliability was satisfactory, with high internal consistency coefficients (ω = 0.93/α = 0.93). Measurement invariance across gender was confirmed, with no significant differences between males and females in jaw function limitation scores. Greater jaw function limitation was significantly associated with increased insomnia, psychological distress, and neck disability, supporting the scale’s convergent validity. **Conclusions**: This study provides a brief, valid, and reliable Arabic version of the 8-item JFLS for assessing jaw and masticatory functional limitations in Arabic-speaking populations. The scale has potential applications in clinical assessment, intervention planning, and cross-cultural research.

## 1. Introduction

Normal jaw function is a key component of oral and overall quality of life. It involves normal movement of the mandible for essential daily activities such as chewing, speaking, yawning, swallowing, breathing, and facial expressions, performed without any pain or restriction in movement and without asymmetry [1]. Such normal movements and functions result from coordinated interaction between the temporomandibular joint, masticatory muscles, and occlusal alignment [2]. In contrast, jaw function limitation (JFL) is defined as a disability or restriction in the capacity to perform normal jaw activities [3]. JFL is common among the general population, especially in certain conditions such as temporomandibular disorders (TMDs) [4]. TMDs affect a significant proportion of the population; a recent meta-analysis estimated the global prevalence at about 29.5%, with female predominance representing a 1.75-fold greater likelihood of being affected. Additionally, TMDs can affect both adolescents and adults. A systematic review showed that the overall prevalence of TMD was approximately 31% for adults/elderly and 11% for children/adolescents [5]. In Arab countries, TMDs were also found to be highly prevalent; 73.4% of postgraduate Egyptian students were affected ranging from mild to severe presentations [6]. In Lebanon, the prevalence has been reported between 17.8% [7] and 19.7%, with a female predominance [8].

Moreover, jaw dysfunction has been found to be frequent in people suffering from degenerative joint disease such as osteoarthritis [9], young people and adolescents suffering from juvenile idiopathic arthritis [10], people with poor dentition [11], and people on some medications (most commonly bisphosphonate due to the risk of mandibular osteonecrosis) [12].

JFL has been associated with poor sleep quality, which appears to act both as a contributing factor to its pathogenesis and as a direct consequence of the condition [13, 14]. Likewise, jaw pain and dysfunction have been shown to have a bidirectional relationship with insomnia as well, particularly in patients with TMD, where worse sleep quality is linked to greater pain severity [15, 16]. Studies reported that people suffering from jaw dysfunction have a higher risk of migraine [17] and more frequent headaches compared with healthy individuals [18]. In addition, Ünlüer *et al*. [19] found a strong correlation between neck disability and JFL, suggesting that greater jaw impairment is closely related to increased neck disability. Furthermore, a meta-analysis and systematic review conducted by Saini *et al*. [20] showed a significant statistical correlation between conditions that involve jaw pain/dysfunction and anxiety, depression and stress. As a matter of fact, JFL was also connected to other psychiatric disorders like schizophrenia [21], especially in patients with TMDs, which has been explained by either the long-term use of some antipsychotics like Haloperidol or the disease itself [22].

### 1.1 Measuring jaw function limitation

To assess jaw function limitation and dysfunction, several scales have been used in the literature. Some examples include the 17-item Mandibular Function Impairment Questionnaire (MFIQ) [23], the 10-item Chewing Function Questionnaire (CFQ) [24], the 49-and 14-item Oral Health Impact Profile (OHIP) [25], the 26-item Quality of Masticatory Function Questionnaire (QMFQ) [26]. However, these scales have several limitations. For instance, the MFIQ only focuses on mastication and jaw movements, without considering other jaw functions like yawning, speaking, and social and emotional communication. The same applies to the CFQ and QMFQ, which assess chewing performance only, and therefore are not reliable tools for adequately measuring all aspects of jaw dysfunction. As for the OHIP, it is widely used to measure overall oral health-related quality of life [25], but it includes a large number of items, making the assessment highly time-consuming. Additionally, this scale lacks specificity for jaw limitation and changes in jaw function since it focuses on multiple domains, such as pain, psychological discomfort, and other parameters.

To address these limitations, the Jaw Function Limitation Scale (JFLS) has been developed to assess specific jaw function limitations, particularly primary and objective limitations in daily activities, such as chewing, jaw mobility, and verbal communication [27]. More precisely, this tool includes three dimensions: vertical jaw mobility, mastication, and verbal/emotional communication. Both the 8- and 20-item scales have shown strong psychometric performance, including strong construct validity, good internal consistency (Cronbach’s α = 0.87 for the 8-item version and 0.95 for the 20-item version), and high test-retest reliability across diverse populations. Its specificity supports targeted clinical interventions and allows precise monitoring of disease progression and treatment outcomes. Beyond TMDs, the JFLS has been used across many other disorders, including primary Sjogren’s syndrome, burning mouth syndrome, skeletal malocclusion, and even healthy individuals [27]. The JFLS is widely used and validated in many countries; it was originally developed in English [27], and later translated and validated in Sweden [3], Croatia [28], China [29], Spain [30], Brazil [31], Turkey [32, 33], and Malaysian English [34].

### 1.2 The present study

In the current study, the JFLS-8 was selected instead of the full 20-item version based on methodological and practical considerations. First, the JFLS-8 has been validated and shown to be reliable in its original form, retaining the core psychometric properties, factor structure, and construct validity of the JFLS-20, making it an appropriate tool for assessing functional limitations of the masticatory system [27]. Moreover, being more concise than the JFLS-20 and more practical for use in busy clinical settings, the JFLS-8 reduces respondent burden and improves data quality, which is particularly important in general-population studies where longer instruments may increase fatigue and reduce completion rates [35]. Furthermore, the JFLS-8 was recommended by the Diagnostic Criteria for Temporomandibular Disorders (DC/TMD) to measure jaw dysfunction during various actions [36], underscoring its international recognition and scientific credibility and making it one of the most widely used tools in the assessment of jaw dysfunction [22].

To the best of our knowledge, no validated Arabic version of the JFLS exists in the literature. The absence of a validated Arabic version represents a significant gap because both linguistic and cultural norms may vary between countries and influence how functional impairment and pain are interpreted by respondents [37]. Therefore, developing a validated Arabic version of this scale would represent substantial progress in assessing jaw dysfunction among Arabic-speaking populations. Moreover, a culturally adapted Arabic JFLS would ensure that the scale maintains its psychometric integrity while accurately portraying the personal experiences, communication styles, and dietary habits unique to Arabic communities. Additionally, having a validated scale in Arabic would harmonize global standards in jaw dysfunction assessment, enabling data comparability, supporting multinational research, and enhancing cross-cultural meta-analyses. The prevalence of TMDs in Lebanon is high, with reports reaching up to 20% of the general Lebanese population [8]. A validated scale would, therefore, facilitate research by enabling epidemiological studies to be conducted on jaw disorders and clarifying their clinical significance and associated burden on the Lebanese population.

The aim of this study was to translate and validate the Arabic JFLS-8 within the general population. Specifically, we aimed to explore its psychometric properties, including construct validity, internal consistency, and convergent validity with measures of insomnia, neck disability, and psychological distress. Thus, we hypothesized that this scale would demonstrate good validity, high internal reliability, and strong convergent validity with insomnia, neck disability and psychological distress.

## 2. Methods

### 2.1 Study design and participants

This cross-sectional study was conducted between August and October 2025. Participants were recruited using a snowball sampling technique via online platforms. The online questionnaire administered to participants was developed using Google Forms and prepared in Arabic. The first part included an online consent checkpoint to confirm voluntary participation and to explain the study adherence to ethical standards such as confidentiality and anonymity. Additionally, it provided an introduction to the study and instructions on how to fill the rest of the survey. Participants eligible for this survey were Lebanese citizens aged 18 years and older, who could speak and read standard Arabic, who had access to the Internet, and provided consent to participate. Exclusion criteria consisted of individuals who did not meet inclusion criteria, or those who did not provide consent. No additional exclusion criteria were applied, ensuring a broad representation of the general population and allowing assessment of a sample reflective of a real-world community setting. Six individuals were excluded because they did not give consent and therefore did not fill in the questionnaire, leaving a final total sample of 427 participants.

### 2.2 Measures

Sociodemographic information was collected, including age, sex, education, marital status, and socioeconomic status. Another part of the questionnaire contained five measures:

Jaw function limitation scale (JFLS-8): This scale was designed to measure global functional limitation of the jaw [27]. In this study, the 8 items scale was used consisting of a three-factor structure: mastication, vertical jaw mobility, and verbal and emotional expression. Higher scores correlate with greater functional limitation.

Insomnia severity index (ISI-7) [38]: This scale is used to screen for insomnia among the general population and to estimate its severity. This index consists of seven items, each item on a Likert scale, and higher scores indicate more severe insomnia. This scale was validated in Arabic [39] with a Cronbach’s alpha of 0.82 and a McDonald’s ω of 0.82. In the current study, Cronbach’s alpha was 0.78.

Neck disability index (NDI-10) [40]: This self-administered questionnaire assesses neck pain and its impact on everyday activities and quality of life. It includes ten items each scored on a 6-point Likert scale ranging from no disability to complete disability. This tool was validated in Arabic and demonstrated a two-factor structure, with a Cronbach’s alpha of 0.89 [41]. In the current study, Cronbach’s alpha was of 0.83.

Patient Health questionnaire-4 (PHQ4) [42]: The PHQ-4 is an ultra-brief screening tool for anxiety and depression comprising two depression items and two anxiety items; higher scores indicate greater symptoms. It was validated in Arabic in Lebanon and showed good internal validity, with both Cronbach’s alpha and a McDonald’s omega equal to 0.86 [43]. In the present study, Cronbach’s alpha was 0.84.

### 2.3 Sample size calculation

A minimal sample size of 160 was calculated based on the guideline of 20 participants per item. Sample size determination followed established recommendations for confirmatory factor analysis, which suggests a participant-to-item ratio of 10:1 to 20:1. We applied the more conservative threshold of 20 participants per item to ensure adequate statistical power, stable parameter estimates, and robust model fit evaluation [44, 45]. Since the scale was composed of 8 items, at least 160 participants were required. This criterion was met with our final sample of 427 participants, thereby strengthening methodological rigor.

### 2.4 Statistical analysis

All questionnaire items were mandatory, and participants were required to complete them before submission, therefore, we had no missing data. We conducted a confirmatory factor analysis in R (lavaan). We used the Maximum Likelihood (ML) estimator because the Weighted Least Squares Mean and Variance Adjusted (WLSMV) estimator is not appropriate when an item has no responses in a given category. WLSMV requires that every category of an ordinal item contains at least some responses in order to estimate threshold and polychoric correlations. When a category is empty, these parameters cannot be reliably computed, which may lead to unstable results. ML, in contrast, can handle sparse or empty categories more flexibly by estimating the model directly from the covariance matrix without relying on threshold estimation. Model fit was assessed using several indices, including Standardized Root Mean Squared Residual (SRMR), root mean square error of approximation (RMSEA), Tucker-Lewis Index (TLI), and comparative fit index (CFI). Adequate model fit was defined as SRMR ≤0.05, RMSEA ≤0.08, and CFI/TLI ≥0.90 [46].

Furthermore, multi-group CFA was applied on the full dataset to test measurement invariance across sex [47]. We assessed measurement invariance across sex for the model using a four-step sequence—configural, metric, scalar, and strict—and reported ΔCFI, ΔRMSEA, ΔSRMR between successive steps. Invariance decisions followed common criteria (ΔCFI ≤0.010, ΔRMSEA ≤0.015, and/or ΔSRMR ≤0.010) [48, 49]. Group differences in JFLS scores were examined with the Mann-Whitney test.

Internal consistency was estimated using Cronbach’s α and McDonald’s ω. Because multivariate normality was not confirmed via Mardia’s skewness (=3645.60; *p * < 0.001) and kurtosis (=119.53; *p * < 0.001), validity evidence was explored through Spearman correlations between the JFLS scale and other constructs.

## 3. Results

Participants’ details are summarized in Table 1.

**Table 1.** Sociodemographic and clinical characteristics of the participants (n = 427).

Variables		n (%) or Mean ± SD
Sex		
	Males	188 (44.0%)
	Females	239 (56.0%)
Education level		
	Primary	8 (1.9%)
	Secondary	24 (5.6%)
	University	395 (92.5%)
Marital status		
	Single	345 (80.8%)
	Married	69 (16.2%)
	Divorced	7 (1.6%)
	Widowed	6 (1.4%)
Employment		
	Full-timer	163 (38.2%)
	Part-timer	45 (10.5%)
	Unemployed	18 (4.2%)
	Student	201 (47.1%)
Age (yr)		27.52 ± 10.61
Household crowding index (person/room)		0.99 ± 0.42

*SD: standard deviation.*

### 3.1 Confirmatory factor analysis

Fit indices of the three-factor model were acceptable (CFI = 0.987; TLI = 0.979; RMSEA = 0.143, 90% Confidence Interval (CI) [0.123–0.163], SRMR = 0.038). Same applies for the fit indices of the second-order model, which was conducted following high correlation coefficients between factors (CFI = 0.987; TLI = 0.979; RMSEA = 0.143, 90% CI [0.123–0.163], SRMR = 0.038) (Fig. 1). Internal reliability was satisfactory for the total score (ω = 0.926/α = 0.926) with a 95% CI of 0.915–0.936 for both values.

**Fig. 1.** Standardized loading factors of the first- and second-order models of the Arabic version of the JFLS items scales via confirmatory factor analysis. JFLS: Jaw Function Limitation Scale.

### 3.2 Measurement invariance

Invariance was shown across both sexes at all levels as verified by the fit indices differences in Table 2. No significant difference in JFLS scores was found between females (Median = 0, IQR = 4) and males (Median = 0, IQR = 4), U = 21068.50, *Z* = −1.20, *p* = 0.230, Effect size = 0.06.

**Table 2.** Measurement invariance of the JFLS items scales across sexes: fit indices and model comparisons.

Model	CFI	RMSEA	SRMR	Model Comparison	ΔCFI	ΔRMSEA	ΔSRMR
Configural	0.993	0.107	0.037				
Metric	0.993	0.095	0.045	Configural *vs.* metric	<0.001	0.012	0.008
Scalar	0.995	0.073	0.037	Metric *vs.* scalar	0.002	0.022	0.008

*CFI: Comparative fit index; RMSEA: root mean square error of approximation; SRMR: Standardized root mean square residual.*

### 3.3 Concurrent validity

Higher JFLS scores were significantly associated with more insomnia, increased psychological distress, and more neck disability (Table 3).

**Table 3.** Correlation matrix of the study variables.

	1	2	3	4	5	6
1. JFLS total	1					
2. JFLS mastication	*Rho* = 0.82; *p * < 0.001	1				
3. JFLS vertical jaw mobility	*Rho* = 0.86; *p * < 0.001	*Rho* = 0.66; *p * < 0.001	1			
4. JFLS verbal and emotional expressions	*Rho* = 0.90; *p * < 0.001	*Rho* = 0.67; *p * < 0.001	*Rho* = 0.76; *p * < 0.001	1		
5. Insomnia severity	*Rho* = 0.23; *p * < 0.001	*Rho* = 0.20; *p * < 0.001	*Rho* = 0.17; *p * < 0.001	*Rho* = 0.20; *p * < 0.001	1	
6. Psychological distress	*Rho* = 0.28; *p * < 0.001	*Rho* = 0.24; *p * < 0.001	*Rho* = 0.26; *p * < 0.001	*Rho* = 0.28; *p * < 0.001	*Rho* = 0.40; *p * < 0.001	1
7. Neck disability	*Rho* = 0.41; *p * < 0.001	*Rho* = 0.34; *p * < 0.001	*Rho* = 0.36; *p * < 0.001	*Rho* = 0.37; *p * < 0.001	*Rho* = 0.52; *p * < 0.001	*Rho* = 0.49; *p * < 0.001

*Numbers in the table reflect Spearman correlation coefficient (Rho). JFLS: Jaw Function Limitation Scale.*

## 4. Discussion

The current cross-sectional study involving 427 participants aimed to evaluate the psychometric properties of the Arabic version of the 8-item JFLS among the Lebanese general population. The main purpose was to evaluate its reliability, validity, and factorial structure compared to the original version. The findings showed that the scale exhibited satisfactory properties, including good internal consistency and adequate construct validity.

CFA of the Arabic version indicated a three-factor structure with acceptable fit indices, consistent with the structural pattern observed in the original study [27]. Previous findings on factor structure of the JFLS among other studies also reported a three-factor structure for the Malaysian English version [34], the Swedish [3], and the Turkish version [33]; however, the Brazilian Portuguese version indicated that more studies are necessary to determine an adequate factor structure for their adaptation of the JFLS [31]. The evidence that the factor loadings exceeded the minimum suggested cutoff of 0.30 [50], and that they were all higher than 0.70, indicates that each of the 8 items of the first- and second-order models of the Arabic version of the JFLS demonstrates high reliability, and thus are considered excellent indicators and contributors to the overall utility of the scale construct.

An additional consideration is that differences in the factor structure found for the Arabic JFLS compared with the other language versions might be partially related to cultural and linguistic dimensions. Previous studies have demonstrated that pain perception and reporting differ significantly between cultures since societal norms shape how people perceive and report functional limitations as well as discomfort [51]. Psychometric evidence from an Arabic translation of the Pain Sensitivity Questionnaire also suggests that linguistic nuances and specific ethnic contexts could change the way items are perceived, even when semantic equivalence has been carefully preserved [52]. These observations support the view that cultural norms concerning stoicism, help-seeking behaviors, and the meaning of jaw-related functional limitations in Arabic-speaking populations might be contributing to the structural differences obtained in the current study.

Although the CFI, TLI, and SRMR values indicated good model fit, the RMSEA value exceeded the commonly accepted threshold (0.08). It is well established that RMSEA can become inflated in models with low degrees of freedom (*df*) [53, 54]. Our model had a small *df* (=17), a condition under which RMSEA tends to overestimate misfit and behave less reliably than other fit indices. Given that the other fit indices (CFI, TLI, and SRMR) met conventional criteria and factor loadings were strong, we interpreted the model as acceptable.

The Arabic version of the JFLS-8 showed excellent internal consistency with Cronbach’s alpha of 0.93, which is higher than the original validation study (α = 0.87) [27], reflecting strong coherence among scale items. However, not measured in the present research, the original study reported an intraclass correlation coefficient (ICC) of 0.93, indicating excellent stability over a 2-week period [27]. High internal consistency was also demonstrated in other translated versions of the JFLS, such as the Turkish (α = 0.91) [33], Chinese (α = 0.91) [29], Spanish (α = 0.95) [30], and the Malaysian English (α = 0.95) [34] versions.

As expected, the total JFLS score strongly and positively correlated with its three subdomains, confirming the internal consistency of the scale. The results also supported convergent validity, with the JFLS scores correlating positively with psychological distress. These findings align with results of other studies and meta-analyses in the literature showing important statistical correlations between disorders comprising jaw pain/dysfunction and depression, stress, and anxiety [20, 55]. Similarly, individuals suffering from TMDs, and consequently jaw dysfunction, were found to have a three-fold higher risk of developing depressive disorders and a seven-fold higher risk of developing anxiety disorders [56]. Conversely, people with a history of major depressive disorder and anxiety disorder were associated with a 5.8-fold and 8.29-fold increased risk of developing TMDs, respectively [56]. In addition, JFL also correlated positively with neck disability, a finding highlighted by Olivo *et al*. [57], who showed that greater jaw impairment—as measured by the JFLS—was closely related to increased cervical impairment, as measured by the NDI scale. Furthermore, it was reported that treatment of jaw dysfunction in TMDs is markedly associated with a reduction in cervical spine pain [58, 59], and that cervical rehabilitation alone or in combination with neck exercises is likely to improve multiple pain outcomes in patients with TMDs [60], highlighting a bidirectional relationship between jaw function limitation and neck disability [57]. Finally, JFL correlated positively with insomnia severity, aligning with previous evidence that higher jaw function limitation leads to increased sleep disturbances, poorer sleep quality, and more severe insomnia [61].

### 4.1 Practical implications

The Arabic version of the JFLS-8 demonstrated excellent psychometric properties, including high reliability and validity among our sample of Lebanese adults. Validating this scale in Arabic constitutes a significant advancement, both for clinical practice and orofacial pain research. A validated measure of JFL would also facilitate standardized assessment of outcomes across different languages and cultures, enhancing cross-national research and collaboration.

Furthermore, in a population where TMDs are prevalent, a diagnostic tool like JFLS-8 would be of great help for clinicians in their daily work. Being short and concise, this self-reported questionnaire consisting of only 8 items offers a significant advantage by reducing patient response burden and facilitating rapid yet comprehensive assessment. This makes it an excellent screening tool among the general population for jaw dysfunction in routine practice, enhancing diagnostic precision, optimizing treatment monitoring, and eventually contributing to better quality of care for individuals suffering from jaw dysfunction-related conditions.

However, while the present study established that the JFLS-8 is a reliable and valid tool for assessing jaw functional limitations in a Lebanese general population sample, further research is warranted. Additional projects are needed to establish the generalizability and applicability of the scale in different contexts. Moreover, future work should confirm its applicability in specific subgroups, such as TMD patients and individuals with chronic jaw pain, as well as validate its usefulness in clinical settings. Longitudinal studies could assess the scale’s responsiveness to temporal changes and evaluate its sensitivity and utility following therapeutic interventions. Comparative studies with longer instruments or TMD-specific questionnaires may also clarify its relative efficiency and precision. Such investigations would strengthen the evidence base for JFLS-8 and guide its application in both research and clinical practice.

### 4.2 Study limitations

This study underscores some limitations that should be acknowledged. First, using a snowball sampling method may introduce selection bias since participants often recruit others from overlapping social groups. Moreover, sampling via online platforms may have also limited representativeness since participants with higher digital literacy or interest in orofacial health may have been more likely to participate, potentially skewing the sample towards younger and more educated participants. Additionally, the mean age of participants in this study leaned towards younger adults making older individuals less represented and limiting the generalizability of the findings. Second, all data were self-reported via an online survey, making the responses subject to some recall bias and social desirability bias. Third, being a cross-sectional study, data was collected at a single point in time, limiting our ability to examine the test-retest reliability, predictive validity, and causality between jaw function limitations and potential contributing factors, such as pain intensity and psychological distress. Moreover, a potential limitation of the current study is that the questionnaire did not assess whether participants had a prior diagnosis of TMD, or if they were suffering from chronic jaw and neck pain, potentially introducing confounding bias. Finally, this validation study was conducted exclusively on a Lebanese sample, limiting generalizability to other Arab countries. This highlights the need for future studies in other Arabic-speaking countries using larger and more demographically diverse samples to examine cross-cultural validity and further strengthen the external validity of the Arabic JFLS-8.

## 5. Conclusions

In conclusion, this study successfully validated the Arabic version of the 8-item JFLS in a Lebanese population, demonstrating strong psychometric properties and confirming it as a valid, reliable, and highly efficient tool for diagnosing and screening jaw dysfunction among specific populations. Its brevity enhances its practicality in both clinical and research settings, allowing rapid assessment without compromising measurement accuracy. Finally, this scale contributes to the global effort toward standardized assessment of jaw functions across Arabic-speaking populations. Further research is, however, highly encouraged in other Arabic-speaking countries.
